# Prognostic stratification of patients with advanced renal cell carcinoma treated with sunitinib: comparison with the Memorial Sloan-Kettering prognostic factors model

**DOI:** 10.1186/1471-2407-10-45

**Published:** 2010-02-18

**Authors:** Aristotelis Bamias, Alexandra Karadimou, Sofia Lampaki, George Lainakis, Lia Malettou, Eleni Timotheadou, Kostas Papazisis, Charalambos Andreadis, Loukas Kontovinis, Ioannis Anastasiou, Kostas Stravodimos, Ioannis Xanthakis, Andreas Skolarikos, Christos Christodoulou, Kostas Syrigos, Christos Papandreou, Evangelia Razi, Urania Dafni, George Fountzilas, Meletios A Dimopoulos

**Affiliations:** 1 Dept of Clinical Therapeutics, Athens University, Medical School, Athens, Greece; 2 Department of Medical Oncology, "Papageorgiou" Hospital, Aristotle University of Thessaloniki, School of Medicine, Thessaloniki, Greece; 3 Data Office, Hellenic Cooperative Oncology Group, Athens, Greece; 4 3rd Dept of Medical Oncology, Theagenion Cancer Hospital, Thessaloniki, Greece; 5 Urology Dept, Athens University, Medical School, Athens, Greece; 6 2nd Dept of Medical Oncology, Metropolitan Hospital, Athens, Greece; 7 3rd Dept of Internal Medicine, University of Athens, Medical School, Athens, Greece; 8 Dept of Medical Oncology, Larisa University Hospital, Larisa, Greece; 9 1st Dept of Medical Oncology, Hygeia Hospital, Athens, Greece

## Abstract

**Background:**

The treatment paradigm in advanced renal cell carcinoma (RCC) has changed in the recent years. Sunitinib has been established as a new standard for first-line therapy. We studied the prognostic significance of baseline characteristics and we compared the risk stratification with the established Memorial Sloan Kettering Cancer Center (MSKCC) model.

**Methods:**

This is a retrospective analysis of patients treated in six Greek Oncology Units of HECOG. Inclusion criteria were: advanced renal cell carcinoma not amenable to surgery and treatment with Sunitinib. Previous cytokine therapy but no targeted agents were allowed. Overall survival (OS) was the major end point. Significance of prognostic factors was evaluated with multivariate cox regression analysis. A model was developed to stratify patients according to risk.

**Results:**

One hundred and nine patients were included. Median follow up has been 15.8 months and median OS 17.1 months (95% CI: 13.7-20.6). Time from diagnosis to the start of Sunitinib (<= 12 months vs. >12 months, p = 0.001), number of metastatic sites (1 vs. >1, p = 0.003) and performance status (PS) (<= 1 vs >1, p = 0.001) were independently associated with OS. Stratification in two risk groups ("low" risk: 0 or 1 risk factors; "high" risk: 2 or 3 risk factors) resulted in distinctly different OS (median not reached [NR] vs. 10.8 [95% confidence interval (CI): 8.3-13.3], p < 0.001). The application of the MSKCC risk criteria resulted in stratification into 3 groups (low and intermediate and poor risk) with distinctly different prognosis underlying its validity. Nevertheless, MSKCC model did not show an improved prognostic performance over the model developed by this analysis.

**Conclusions:**

Studies on risk stratification of patients with advanced RCC treated with targeted therapies are warranted. Our results suggest that a simpler than the MSKCC model can be developed. Such models should be further validated.

## Background

Renal cancer is the third most frequent malignancy of the urinary tract and accounts for 3% of all adult malignancies [1]. Most patients (70-80%) presenting with localized disease can be cured with surgery. On the contrary, advanced disease or relapses after radical nephrectomy is usually incurable. In total, nearly 50% of patients with renal cell carcinoma will present with or develop metastatic disease [1,2]. Prognosis in patients with advanced disease remains poor and 5-year life expectancy is less than 20% [2,3].

The cytokines Interleukin-2 (IL-2) and Interferon-α (IFN-α) have been the standard of care in metastatic RCC for more than fifteen years. This treatment achieves low response rates, duration of response is usually short and long-term survival is rare, while toxicity is considerable [4,5]. In spite of the above limitations, some patients will benefit from cytokine treatment. Retrospective analyses and the recently reported PERCY Quattro trial [6] identified certain characteristics, which allow for the selection of patients likely to benefit from this treatment: LDH, Karnofsky PS, nephrectomy, time from nephrectomy, calcium and hemoglobin levels have been associated with independent prognostic significance [6-9]. The combination of these factors led to the development of a prognostic model by the MSKCC including three patient groups with a statistically significant and, more importantly, clinically relevant difference in survival [8]. This model was subsequently validated independently [10] and proved valuable in selecting patients likely to benefit from cytokine therapy and in interpreting results of phase II and III studies.

Recent advances in our understanding of the biology of RCC and especially the role of angiogenesis in the development and expansion of this tumor led to the development of novel targeted therapies [11-13], which proved to be superior to interferon. Sunitinib is an inhibitor of the split-kinase-domain family of receptor tyrosine kinases (including Vascular endothelial growth factor-VEGF) [14]. Its antitumour activity results from inhibition of angiogenesis through blockade of the endothelial cell VEGF pathway and PDGFR-β expression in pericytes but also tumour cell proliferation [15]. It has been recently established as first-line treatment for advanced RCC, following the results of a randomised phase III trial, which showed a significant advantage over interferon-a in progression-free survival (PFS) [11]. In spite of this undisputed benefit, the prognosis of advanced RCC remains poor, while the toxicity of sunitinib (as well as that of other novel agents) is considerable [16]. There is, therefore, a need to select patients likely to benefit from these therapies.

In contrast to cytokines, data on prognostic and predictive factors during treatment with sunitinib are limited. The MSKCC model has been used for the design of all phase III trials using modern therapies. Nevertheless, there may be limitations associated with its use in this context. This model was developed with patients undergoing treatment with cytokines. Although it could be argued that the factors used in this model reflect the biological behavior of the disease, and, therefore, may be applicable to any therapy, its utility in the context of targeted therapies has not been fully evaluated. Furthermore, all randomized studies mainly included patients with low or intermediate risk, i.e. populations with different composition than that of the population used to develop the MSKCC model. Finally, the MSKCC model has been validated as a predictor of OS, while PFS has been the major end point in all randomized trials testing targeted therapies. For the above reasons we analysed the advanced RCC database of HECOG in order to study prognostic clinicopathological factors in patients treated with sunitinib. We also compared the prognostic accuracy of the model, which was produced with that of the MSKCC model in our population.

## Methods

### Patients

Since January 2008, clinicopathological data of patients with advanced RCC treated with targeted agents have been entered prospectively into a database of HECOG. Data for patients treated with sunitinib after its approval as first-line therapy in metastatic RCC in Greece (end of 2006) and prior to 2008 were retrieved from their medical records and were entered retrospectively. Patients had given their written permission for the retrieval of data from their medical records for research purposes prior to the initiation of treatment. All patients were homogenously treated and followed-up according to a protocol, which was based on the approved SPC and the published clinical studies. Sunitinib was administered at the approved dose of 50 mg daily on a 4 weeks on-2 weeks off schedule. Treatment was interrupted in case of Grade 3 or 4 toxicity and was reintroduced when toxicity was ≤ Grade 1. In case of Grade 3 non haematological toxicity or Grade 4 haematological toxicity, there was a successive reduction at a daily dose of 37.5 mg and 25 mg. Thyroid dysfunction and arterial hypertension were managed with appropriate medication without dose reductions. Tumor evaluation was performed every 2-3 cycles of treatment unless clinically indicated.

This is a retrospective analysis of patients treated with sunitinib in six Greek Oncology Units of HECOG. Inclusion criteria were: advanced RCC not amenable to surgery and treatment with sunitinib. Adjuvant or first-line treatment with interferon was allowed but no previous targeted therapy with sorafenib, bevacizumab or temsirolimus. Measurable or evaluable disease was not required for inclusion in the analysis. Data was frozen at April 2009.

### Statistical analysis

The SPSS software was used for statistical analysis (SPSS for Windows, version 15.0, SPSS Inc.). OS was measured from the date of randomization until death from any cause. PFS was measured from the date of randomization until objective tumor progression or death. Time-to-event distributions were estimated using Kaplan-Meier curves. The Cox proportional hazards model was used to assess the relationship of OS with various clinical and laboratory variables. The backward selection procedure with removal criterion p > 0.10 based on Likelihood ratio test was performed to identify significant variables among the following: number of metastatic sites (1 vs >1), time between diagnosis/surgery and sunitinib initiation (<= 12 months vs >12 months), ECOG PS (<= 1 vs >1), Haemoglobin (<13 g/dL vs >= 13 g/dL [Male]; <11.5 g/dL vs >= 11.5 g/dL [Female]), Calcium (<10 vs >= 10), Lactate dehydrogenase (normal vs abnormal), Alkaline phosphatase (normal vs abnormal), prior nephrectomy (no vs yes).

A model was developed to stratify patients according to risk. Significant risk factors were identified and model selection was performed through Likelihood ratio tests, comparing models to the established MSKCC models in the literature [8,9]. The prognostic performance of the models was assessed through ROC curves, and AUC comparison was performed by a non-parametric test proposed by DeLong [17]. STATA (version 10) was used for the analysis.

## Results

### Patient characteristics

One hundred and nine patients (M: 80, F: 29; median age 59) were included in this analysis. Their characteristics are shown in Table 1. Seventeen patients (15%) had been treated with IFNa 2a, while 86 (79%) had undergone nephrectomy. One hundred patients (91%) had clear-cell carcinoma, while in another 3 (3%) a clear-cell component with other elements was also detected. The remaining cases were pure non clear-cell carcinomas. At the time of analysis a total of 724 cycles of Sunitinib had been administered (median 5, range 1-35).

**Table 1:** Characteristics of the 109 patients included in the analysis

Age (Median, range)	59	30-79
	**n**	**(%)**

Sex		
Male	80	(73)
Female	29	(27)

Nephrectomy		
No	23	(21)
Yes	86	(79)

Time between diagnosis and Sutent initiation		
<= 12 months	53	(49)
>12 months	54	(49)
Unknown	2	(2)

Histology		
Clear Cell	100	(91)
Papillary	2	(2)
Chromophobe	2	(2)
Mixed	3	(3)
Unclassified	2	(2)

Performance Status		
0	59	(54)
1	37	(34)
2	13	(12)

Number of Metastatic sites		
1	32	(29)
>1	77	(71)

Site of metastatic disease		
Lung	75	(69)
Nodes	38	(35)
Liver	10	(9)
Renal bed	27	(25)
Bones	39	(36)
Brain	8	(7)

Hb		
Median (range)	12.1	(8.6-17.9)
<13 for Males, <11.5 for Females	57	(52)
>= 13 for Males, >= 11.5 for Females	50	(46)
Unknown	2	(2)

Ca		
Median (range)	9.4	(1.0-12.1)
<10	85	(78)
>= 10	16	(15)
Unknown	8	(7)

LDH		
Normal	58	(53)
Abnormal	38	(35)
Unknown	13	(12)

ALP		
Normal	74	(68)
Abnormal	29	(27)
Unknown	6	(5)

### Tumor response and PFS

Thirty-five patients (32%) were still on treatment. Eighty-nine patients with at least one tumor evaluation were assessable for response. Four patients experienced early death prior to tumor assessment, while in the remaining 16 cases no tumor assessment had been performed at the time of analysis. One patient (1.1%, 95% CI: 0.02-5) had complete tumor response (CR) and 19 patients (21.3%, 95% CI: 13.8-30.1) had a partial response (PR) for a total of 22.4% objective response rate (ORR). Stable disease (SD) was observed in 49 cases (55.1%, 95% CI: 39.4-59.8), while 20 patients had progressive disease at first tumor evaluation (22.5%, 95% CI: 14.2-30.9).

Median follow up was 15.8 months (range for surviving patients 0.1-31.5). Median PFS for the whole cohort was 8.9 months (95% CI: 6.4-11.4), while 1-year PFS rate was 40%.

### Overall survival analyses

**Figure 1:**
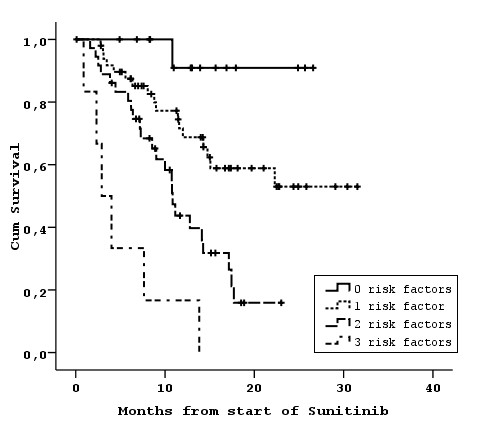
**Overall survival according to risk factors (0-3) found in our analysis**.

Median OS was 17.1 months (95% CI: 13.7-20.6) and 1-year survival rate 61%. During follow up 48 patients (44%) died from RCC. Univariate analysis showed that the following factors were associated with worse OS: >1 metastatic sites (p = 0.004), <= 12 months between diagnosis and treatment with sunitinib (p = 0.001), PS >1 (p = 0.023), abnormal ALP (p = 0.004), low Hb levels (p = 0.035) and no prior nephrectomy (p = 0.001) (Table 2). The effect of LDH levels was not statistically significant (p = 0.063), while previous therapy with IFN or histological type did not affect OS (p = 0.217 and p = 0.222, respectively). The independent association with survival was examined in the backward selection procedure. With a removal criterion of p > 0.10, the final model for predicting survival included three independent risk factors: number of metastatic sites, interval from diagnosis/surgery to treatment initiation, and PS (Table 3). The combination of these three factors resulted in the stratification into 4 groups with distinct separation of OS curves (Table 4, Figure 1).

**Table 2:** Univariate Cox regression (OS)

	Median OS (95% CI)	HR (95% CI)	p
Metastatic sites			0.004
1	NR	1	
>1	13.8 (10.4-17.3)	3.55 (1.50-8.36)	

Time between diagnosis/surgery and Sunitinib initiation			0.001
<= 12 months	11.6 (8.2-14.9)	1	
>12 months	NR	0.36 (0.19-0.66)	

Performance Status			0.023
<= 1	17.4 (10.2-24.6)	1	
>1	7.6 (0-18.5)	2.32 (1.12-4.80)	

Hb			0.035
<13 for Males, <11.5 for Females	14.3 (11.3-17.3)	1	
>= 13 for Males, >= 11.5 for Females	NR	0.51 (0.28-0.95)	

Ca			0.538
<10	17.4 (8.3-26.6)	1	
>= 10	15.1 (8.4-21.7)	1.29 (0.57-2.90)	

LDH			0.063
Normal	NR	1	
Abnormal	13.8 (8.7-18.9)	1.78 (0.97-3.26)	

ALP			0.004
Normal	22.3	1	
Abnormal	10.7 (6.5-15)	2.46 (1.34-4.50)	

Nephrectomy			0.001
No	8.9 (3.8-14.2)	1	
Yes	22.3	0.36 (0.19-0.65)	

NR: not reached

**Table 3:** Multivariate Cox regression (Survival)

		HR	95% CI	p
Model 1	MSKCC - Risk Group			
	Favourable	1	-	-
	Intermediate	8.06	1.09-59.59	0.041
	Poor	18.21	2.42-136.88	0.005

Model 2	Metastatic sites			
	1	1	-	-
	>1	3.67	1.56-8.66	0.003
	
	Time between diagnosis/surgery and Sunitinib initiation			
	<= 12 months	1	-	-
	>12 months	0.36	0.19-0.67	0.001
	
	Performance Status			
	<= 1	1	-	-
	>1	3.04	1.46-6.32	0.003

Model 3	4-Groups			
	0 risk	1	-	-
	1 risk	5.64	0.75-42.39	0.093
	2 risk	14.62	1.97-108.26	0.009
	3 risk	51.20	6.09-430.42	0.000

Model 4	3-Groups			
	0 or 1 risk	1	-	-
	2 risk	3.26	1.76-6.06	0.000
	3 risk	11.45	4.41-29.74	0.000

Model 5	2-Groups			
	0 or 1 risk	1	-	-
	2 or 3 risk	3.63	2.01-6.57	0.000

**Table 4:** Risk stratification based on the 3 independent prognostic factors found in multivariate analysis (number of metastatic sites, time from diagnosis/surgery and PS) and the MSKCC criteria (LDH, Hb, Ca, PS, time from diagnosis/surgery)

Stratification according to the risk factors identified in the current series								
Risk factors	Pts	Events	Censored (%)	Mean OS (95% CI)	Median OS (95% CI)			
0	15	1	14 (93.3)	25.2 (22.5-27.8)	NR			
1	50	17	33 (66.0)	21.8 (18.2-25.3)	NR			
2	36	24	12 (33.3)	12.1 (9.7-14.4)	10.8 (9.5-12.2)			
3	6	6	0 (0.0)	5.3 (1.4-9.1)	2.9 (0.9-4.9)			
Total	107	48	59 (55.1)	18.7 (16.2-21.2)	17.1 (13.9-20.3)			
**Pairwise Comparison**								
***No of risk factors***	**0**		**1**		**2**		**3**	
	**Chi-Square**	**p**	**Chi-Square**	**p**	**Chi-Square**	**p**	**Chi-Square**	**p**
0			3.680	.055	12.018	.001	22.564	.000
1	3.680	.055			9.368	.002	25.144	.000
2	12.018	.001	9.368	.002			7.969	.005
3	22.564	.000	25.144	.000	7.969	.005		
**Stratification in 2 prognostic groups**								
**Risk factors**	**Pts(%)**	**Events**	**Censored (%)**	**Mean OS (95% CI)**	**Median OS (95% CI)**			
0 or 1	65	18	47 (72.3)	23.5 (20.4-26.5)	NR			
2 or 3	43	30	13 (30.2)	11.3 (9.1-13.4)	10.8 (8.3-13.3)			
Total	108	48	60 (55.6)	18.8 (16.3-21.3)	17.1 (13.9-20.3)			
								
**Stratification according to the MSKCC model**								
Risk factors	Pts	Events	Censored (%)	Mean OS (95% CI)	Median OS (95% CI)			
0 (favorable)	15	1	14 (93.3)	29.9 (26.7-33.0)	NR			
1,2 (intermediate)	56	24	32 (57.1)	19.1 (15.8-22.3)	17.4			
3,4,5 (poor)	25	18	7 (28.0)	10.4 (7.9-12.8)	11.1 (3.9-18.4)			
Total	96	43	53 (55.2)	18.9 (16.3-21.6)	15.1 (12.1-18.1)			
								
**Pairwise Comparisons**								
***No of risk factors***	**0**		**1,2**		**3,4,5**			
	**Chi-Square**	**Sig.**	**Chi-Square**	**Sig.**	**Chi-Square**	**Sig.**		
0 (favorable)			5.953	.015	16.052	.000		
1,2 (intermediate)	5.953	.015			7.081	.008		
3,4,5 (poor)	16.052	.000	7.081	.008				

NR: not reached

### Prognostic stratification and comparison with the MSKCC model

**Figure 2:**
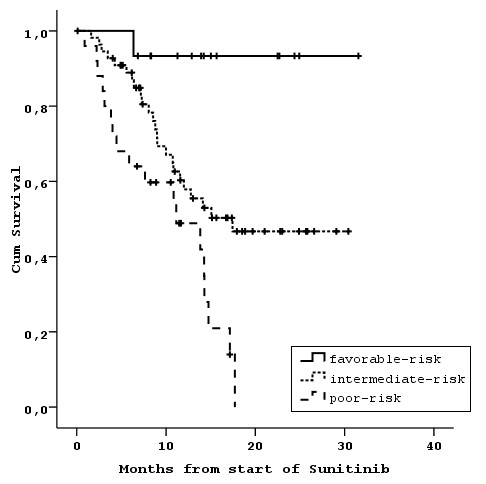
**Overall survival according to the MSKCC criteria**.

The application of the MSKCC model, using stratification by LDH, Hb, Ca, PS, time from diagnosis to initiation of Sunitinib into 3 risk groups (favorable: 0 risk factors, intermediate: 1 or 2 risk factors, and poor: 3, 4 or 5 risk factors) (Model 1), resulted in populations with distinctly separated OS curves (Table 4, Figure 2).

The breakdown of patients and events into the four risk categories based on the identified risk factors and how this corresponds to the breakdown to the three MSKCC categories is presented in Table 5. Among the 15 patients in the favorable risk category by MSKCC (death rate [DR]/year in observation = 0.05), no deaths were observed for the six who were also identified as belonging to the most favorable risk category by the proposed model (DR = 0), while for the 9 who were categorized in the second risk category by the proposed model one of them died (DR = 0.07). Among the 25 patients with poor risk according to Motzer (DR = 0.95), for the patients in the least favorable risk category by both models, all 6 patients died in a short period (DR = 2.22 deaths per year in observation), while for the remaining 19 patients categorized in the two intermediate risk categories according to the proposed model, 12 died during the observation period (DR = 0.74). Among the 55 patients of the intermediate risk according to Motzer (DR = 0.42), the DR according to the proposed stratification was 0.08, 0.38 and 0.72, for the 9 patients without any risk factor, the 26 with one risk factor and the 20 with two risk factors, respectively. Thus, the separation of the intermediate category in the MSKCC model into the first three categories based on the proposed stratification seems to offer better prognostic ability for OS as is the case for the poor and favorable risk categories.

**Table 5:** Breakdown of patients, events, total observation time (in years) and death rate according to the 4 risk category proposed model and the MSKCC model.

Events/Patients		Proposed model				
		
		0 risk	1 risk	2 risk	3 risk	Total
MSKCC model	Favorable risk	0/6	1/9	0/0	0/0	**1/15**
	
	Intermediate risk	1/9	10/26	13/20	0/0	**24/55**
	
	Poor risk	0/0	3/5	9/14	6/6	**18/25**
	
	Total	**1/15**	**14/40**	**22/34**	**6/6**	**43/95**

**Events/Total observation time (in years)^1^**		**Proposed model**				
		
		**0 risk**	**1 risk**	**2 risk**	**3 risk**	**Total**

MSKCC model	Favorable risk	0/5.5	1/14.5	0/NA	0/NA	**1/20**
	
	Intermediate risk	1/12.7	10/26.3	13/18.1	0/NA	**24/57.1**
	
	Poor risk	0/NA	3/5.1	9/11.2	6/2.7	**18/19**
	
	Total	**1/18.2**	**14/45.9**	**22/29.3**	**6/2.7**	**43/96.1**

**Total observation time (in years)^1^** **(Death rate per year in observation)**		**Proposed model**				
	
		**0 risk**	**1 risk**	**2 risk**	**3 risk**	**Total**

MSKCC model	Favorable risk	5.5 (0)	14.5 (0.07)	NA	NA	**20 (0.05)**
	
	Intermediate risk	12.7 (0.08)	26.3 (0.38)	18.1 (0.72)	NA	**57.1 (0.42)**
	
	Poor risk	NA	5.1 (0.59)	11.2 (0.80)	2.7 (2.22)	**19 (0.95)**
	
	Total	**18.2 (0.05)**	**45.9 (0.30)**	**29.3 (0.75)**	**2.7 (2.22)**	**96.1 (0.45)**

^1^Defined as the time up to last contact or death

^2^NA: Not applicable due to non-existing cases in these categories

**Figure 3:**
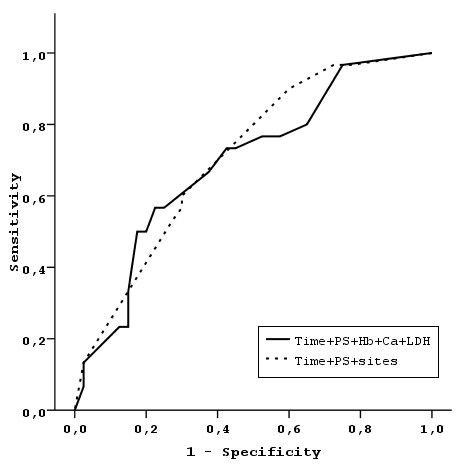
**ROC curves comparing the MSKCC model with the model developed by our analysis**.

**Figure 4:**
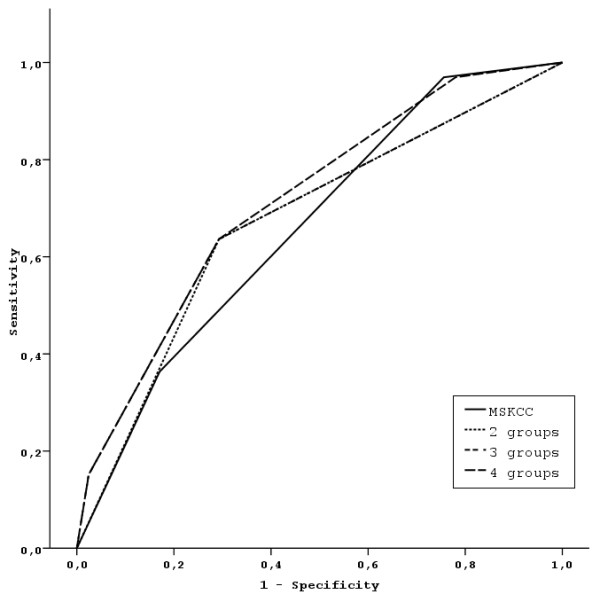
**ROC curves comparing the MSKCC model with the final model resulting after the collapse of four risk groups into two**.

The prognostic ability of the MSKCC model was compared to the models based on the identified three independent risk factors (number of metastatic sites, interval from diagnosis/surgery to treatment initiation and PS). These are the models with the three factors (Model 2), and the models created by the combination of them into 4 categories (number of risk factors present: 0, 1, 2, 3 - Model 3), into 3 categories by collapsing the two more favorable categories into one (Model 4), and finally into 2 categories by additionally collapsing the two less favorable categories into one (Model 5) (Table 3). Multivariate models 1-5 are presented in Table 3. The prognostic ability of the proposed risk stratification into either 4, 3 or 2 different categories were compared to the MSKCC risk stratification. Areas under the Curve (AUCs) for the corresponding ROCs were 0.715, 0.686, 0.672, 0.661 for the 4, 3, 2, and MSKCC risk categories respectively. The sensitivity and specificity of each model were based on the predicted probabilities of the corresponding logistic models at the 12 month follow-up time. No significant differences were found. Figures 3 and 4 shows the resulting ROC curves.

**Figure 5:**
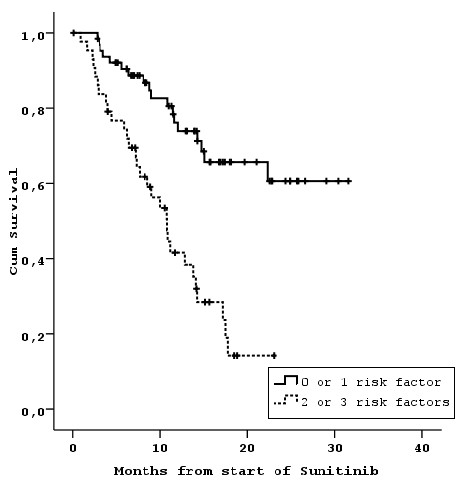
**Overall survival after stratification in low and high risk patients according to the final model**.

The model with stratification into 4 groups (Model 3) seemed to be the more informative from all models proposed (highest AUC = 0.715). Nevertheless, due to lack of statistically significant differences and the small number of patients in each of the worst and best risk categories, the most parsimonious model (Model 5) with only two risk categories (0 or 1 risk factors [n = 65] vs. 2 or 3 risk factors [N = 43], was chosen to be presented here in more detail (Table 4, Figure 5). Descriptive statistics for OS in the 4 prognostic groups and the two prognostic groups after collapsing the risk categories, are presented in Table 4. For the final model 5, the resulting difference in OS between the two risk categories was highly significant (P < 0.001). The hazard ratio for the high-risk group is 3.63 (95% CI: 2.01-6.57) compared to the low-risk group. One-year survival rates for the two prognostic groups were: 74% and 42%, respectively.

Similar analyses (not shown) were also performed substituting nephrectomy for interval from diagnosis/surgery and exploring whether adding metastatic sites in the Motzer model improves it, which was found to be so (p = 0.001). Conclusions were not altered from these analyses.

## Discussion

Selection of patients with metastatic RCC likely to benefit from antiangiogenic therapies represents an unmet medical need. Preferably, a biological surrogate marker which predicts for a favorable response to a targeted agent should be used. At the moment, validated markers do not exist, although certain positive associations have been recently published [18-20]. Until the prospective validation of such markers, selection of patients will rely upon baseline clinicopathological characteristics of patients who are candidates for targeted therapies.

In a retrospective analysis we assessed prognostic factors in a series of 109 patients. These patients have been treated in six Oncology Units in Greece, outside clinical studies, thus accurately reflecting the current clinical practice in advanced RCC in our country. PFS and ORR, based on investigator assessments, have been reported for descriptive purposes. The median PFS of 8.9 months is similar to that reported for expanded access programs (EAP) for sunitinib [21,22], taking into consideration that our population included fewer patients of favorable prognosis, according to MSKCC criteria. ORR is lower than that reported in the randomized study by Motzer et al [11], which is again consistent with the data from EAPs. It should also be noted that 32% of our patients were still on treatment, which may have resulted in underestimation of the ORR, as suggested by a recent analysis showing a higher RR after longer follow up [23]. The above data suggest that our cohort is a representative population treated with Sunitinib worldwide.

Certain limitations should be taken into consideration in relation to this analysis. We included patients previously treated with IFN and with non-clear cell histology (15% and 4% of the total, respectively). Nevertheless, these characteristics were not associated with prognosis, as also shown in an Italian EAP [22]. Finally, the median follow up is fairly short to estimate long-term survival in our cohort. The patients are still on follow up and long-term survival will be reported upon maturation.

Median OS for the whole cohort was 17.1 months. This is somewhat lower than that reported in the two EAPs, probably reflecting the low proportion of favorable prognosis patients (according to the MSKCC criteria) included in our cohort. This is the first analysis of prognostic factors regarding OS in unselected patients treated with sunitinib. Although PFS has become an established end point for assessing new agents in RCC, we believe that OS should still remain the major end point in studying prognostic factors in unselected patients. Especially in retrospective analyses, PFS is based on investigators' assessments and time of efficacy assessment may vary. In addition, the application of RECIST criteria for defining progression may not be adequate in the era of targeted therapies [24]. The use of PFS as a major end point for analysis of prognosis is justified in randomized studies which allow crossover to a more effective therapy, which may have an impact on survival. In our cohort, this concern would be justifiable if patients had received such therapy upon progression on Sunitinib. Although there is evidence that targeted therapies may be effective after the failure of each other, only everolimus has proven prolongation of PFS benefit after Sunitinib [25]. This agent is not yet available in Greece.

The analysis of survival data in unselected patients may be of value for groups, which are underrepresented in large studies, such as poor risk patients according to MSKCC criteria. The value of sunitinib in this group is not clarified. We showed a median OS of 11.2 months in 25 patients of this category. This is a promising result, taking into consideration the median of 5 months shown for IFN [9] and 7 months reported for Temsirolimus [26], which is considered the current standard for these patients. Although these are indirect comparisons, our result supports subgroups analyses performed in the context of a randomized study [11], suggesting that sunitinib is effective in poor risk patients.

We identified time from diagnosis to start of Sunitinib, number of metastatic sites and PS as independent prognostic factors. The prognostic significance of these factors has been previously identified in patients treated with cytokines [8,9], indicating that they are associated with the behavior of the disease rather with a specific form of therapy. The combination of these factors resulted in two groups with statistically and clinically significant difference in outcome. It should be noted that the collapsing of the initial four risk groups (0,1,2 or 3 risk factors) into the final two was largely the result of the relatively small sample size, which represents a limitation of this analysis. Given the heterogeneity of mRCC, separation into more risk groups may be more informative as indeed was suggested by our statistical analysis. For these reasons, we plan to further study and validate our model in larger cohorts of patients. We compared our model with the established MSKCC model. The use of a different therapy from cytokines may have an impact on the prognostic significance of certain factors included in that model. In a recent analysis of the 375 patients receiving first-line sunitinib in the context of the randomized study [11], the same factors plus the presence of bone metastases were found to be prognostically significant for OS [27]. The application of this model to our population resulted in 3 prognostically distinct groups, which underlines its validity. Nevertheless, further improvement may be possible. This model uses 2 clinical factors (time from diagnosis to the start of systemic therapy and PS) and 3 laboratory parameters (corrected calcium, hemoglobin and LDH). The use of laboratory parameters makes retrospective classification of patients with missing data impossible and this might represent an advantage of our prognostic algorithm. More importantly, the distribution of patients according to the MSKCC model is uneven: almost 60% of the population belonged to the intermediate risk group [9,10]. The disproportionately large number of patients in this group suggests that it may be somewhat heterogeneous in respect to outcome. On the contrary, the two groups of our model had a more even distribution. The breakdown of the 55 intermediate risk patients of the MSKCC group according to our model resulted in two groups of 35 and 20 patients with a more than 2-fold difference in the annual death rate, suggesting a clinically meaningful prognostic separation of this group. The comparison of the MSKCC model with our model showed no significant differences. For the above reasons, we believe that further validation of our model is warranted.

## Conclusions

Our study indicates that simple prognostic models, based on clinical factors, may apply to metastatic RCC treated with sunitinib and further studies to validate such models are warranted. These models may be substituted for the widely applied MSKCC model.

## Competing interests

AB has received honoraria by PFIZER, BAYER and NOVARTIS. No other author declared that they have any competing interest.

## Authors' contributions

AB has participated in the conception and the design of the study, acquisition, analysis and interpretation of data and writing the manuscript. AK, SL, GL, ET, KP, CA, LK, IA, KS, IX, AS have made substantial contribution to the acquisition of data and final approval of the manuscript. CC, KS, CP, ER have made substantial contribution to the acquisition of data, revising and final approval of the manuscript LM, UD designed and performed the statistical analysis. GF, MAD have participated in the conception and the design of the study, interpretation of data and critically revising the manuscript. All authors have read and approved the final version of the manuscript.

## Pre-publication history

The pre-publication history for this paper can be accessed here:

http://www.biomedcentral.com/1471-2407/10/45/prepub
